# Associations between a Polymorphism in the Pleiotropic *GCKR* and Age-Related Phenotypes: The HALCyon Programme

**DOI:** 10.1371/journal.pone.0070045

**Published:** 2013-07-23

**Authors:** Tamuno Alfred, Yoav Ben-Shlomo, Rachel Cooper, Rebecca Hardy, Ian J. Deary, Jane Elliott, Sarah E. Harris, Mika Kivimaki, Meena Kumari, Chris Power, John M. Starr, Diana Kuh, Ian N. M. Day, the HALCyon study team

**Affiliations:** 1 School of Social and Community Medicine, University of Bristol, Bristol, United Kingdom; 2 MRC Unit for Lifelong Health and Ageing and Division of Population Health, University College London, London, United Kingdom; 3 Centre for Cognitive Ageing and Cognitive Epidemiology, University of Edinburgh, Edinburgh, United Kingdom; 4 Department of Psychology, University of Edinburgh, Edinburgh, United Kingdom; 5 Centre for Longitudinal Studies, Department of Quantitative Social Sciences, Institute of Education, London, United Kingdom; 6 Medical Genetics Section, University of Edinburgh, Edinburgh, United Kingdom; 7 Department of Epidemiology and Public Health, University College London, London, United Kingdom; 8 MRC Centre of Epidemiology for Child Health/Centre for Paediatric Epidemiology and Biostatistics, University College London Institute of Child Health, London, United Kingdom; 9 Alzheimer Scotland Dementia Research Centre, University of Edinburgh, Edinburgh, United Kingdom; 10 MRC Centre for Causal Analyses in Translational Epidemiology, School of Social and Community Medicine, University of Bristol, Bristol, United Kingdom; University of Illinois at Chicago, United States of America

## Abstract

**Background:**

The glucokinase regulatory protein encoded by *GCKR* plays an important role in glucose metabolism and a single nucleotide polymorphism (SNP) rs1260326 (P446L) in the gene has been associated with several age-related biomarkers, including triglycerides, glucose, insulin and apolipoproteins. However, associations between SNPs in the gene and other ageing phenotypes such as cognitive and physical capability have not been reported.

**Methods:**

As part of the Healthy Ageing across the Life Course (HALCyon) collaborative research programme, men and women from five UK cohorts aged between 44 and 90+ years were genotyped for rs1260326. Meta-analysis was used to pool within-study genotypic associations between the SNP and several age-related phenotypes, including body mass index (BMI), blood lipid levels, lung function, and cognitive and physical capability.

**Results:**

We confirm the associations between the minor allele of the SNP and higher triglycerides and lower glucose levels. We also observed a triglyceride-independent association between the minor allele and lower BMI (pooled beta on z-score = −0.04, p-value = 0.0001, n = 16,251). Furthermore, there was some evidence for gene-environment interactions, including physical activity attenuating the effects on triglycerides. However, no associations were observed with measures of cognitive and physical capability.

**Conclusion:**

Findings from middle-aged to older adults confirm associations between rs1260326 *GCKR* and triglycerides and glucose, suggest possible gene-environment interactions, but do not provide evidence that its relevance extends to cognitive and physical capability.

## Introduction

The ageing process is complex, comprising several mechanisms and resulting in increased frailty and disease susceptibility [1]. Despite there being no single dominant mechanism of ageing, there are several reported associations observed among age-related phenotypes [2]–[5], suggesting phenotypes may directly or indirectly affect others and/or that distinct phenotypes may be affected by common causes [6]. For instance, associations observed between cognitive and physical performance in older adults [2], [4] may be indicative of genetic factors influencing age-related diseases that lead to the impairment of both sets of indicators or of direct genetic effects on the indicators [6]. Whilst not all studies [7] support a common cause hypothesis for ageing phenotypes [8], [9], there are some genes that appear to influence multiple age-related traits.

*GCKR* (glucokinase (hexokinase 4) regulator) is one such gene with potentially pleiotropic effects. Encoding the glucokinase regulatory protein that regulates the activity of glucokinase (GCK), a regulator of glucose, a non-synonymous [10] single nucleotide polymorphism (SNP), rs1260326 (P446L), in the gene appears to be functional, inhibiting GCK activity in the liver [11]. This property has several phenotypic manifestations, with many reports of the T allele of the SNP being associated with increased levels of triglycerides[10], [12]–[14], in addition to increased C-reactive protein (CRP) [15], factor VII [16], apolipoproteins [12], [13], albumin [17], creatinine [18], protein C [19] and uric acid [20], as well as lower levels of insulin [21] and fasting glucose [10], [22] in candidate gene and genome-wide association studies (GWAS). SNPs in strong linkage disequilibrium with the variant have also been associated with serum calcium [23] and risk of Crohn’s disease [24]. Furthermore, these genotypic associations may be environmentally influenced as there is evidence that an intensive lifestyle intervention, including increased physical activity, may reduce the effects of rs1260326 on triglyceride levels [25].

Given the hypothesised relationships between some of these biomarkers and other important age-related phenotypes, such as physical and cognitive performance [5], [26], we hypothesised that *GCKR* may also be relevant to the capacity to undertake the physical and mental tasks of daily living. Indicators of physical capability, including grip strength, decline from mid-life [27] and have been associated with morbidity [28] and mortality [29] rates. The substantial heritability estimates for these indicators [30], [31] suggest genetic variants may contribute to their inter-individual variability. Measures of cognitive capability, such as verbal fluency, also have a substantial genetic component [30], [32] and show associations with mortality rate; though these associations may be partly explained by lifestyle and socioeconomic factors [33], [34]. We conducted analyses on 16,251 participants aged between 44 and 90+ from five UK cohorts as part of the HALCyon research programme (Healthy Ageing across the Life Course; www.halcyon.ac.uk) to investigate associations between rs1260326 (*GCKR*) and several age-related phenotypes. From this multi-cohort exploratory study we provide further evidence for the well-reported associations between the SNP and triglycerides, investigate several novel associations, including those with lung function, physical and cognitive capability, as well as explore interactions between the SNP and physical activity and sex on these measures.

## Methods

### Ethics Statement

Informed consent was obtained from all participants. Ethical approval was obtained from the South-east Multi-centre Research Ethics Committee, the North Thames Multi-centre Research Ethics Committee, the Joint UCL/UCLH Committees on the Ethics of Human Research (Committee Alpha), the Medical Research and Ethics Committee, and the Lothian Research Ethics Committee.

### Study Populations

The National Child Development Study (NCDS) follows individuals from all births in England, Scotland and Wales during 1 week in March 1958. In 2002–04 a Biomedical Survey was conducted during home visits by a research nurse. Following informed consent, DNA was extracted from 8017 participants aged 44–45 years; the sample with immortalised cell line culture (n = 7526) is used here. In 2008–09, an eighth sweep was carried out during which cognitive performance tests were conducted [35]. Further details of the study are available on the cohort’s genetic information website (http://www.b58cgene.sgul.ac.uk/) and elsewhere [36].

The Medical Research Council National Survey of Health and Development (NSHD) comprises participants sampled from all births in a week in March 1946 and followed up since. In 1999, at age 53 years, men and women were visited by a research nurse and consent for DNA extraction was given by approximately 2900 members of the cohort. Details of the data collected and the several phases of the study are available on the cohort’s website (www.nshd.mrc.ac.uk) and elsewhere [37].

The Whitehall II study targeted all civil servants aged between 35 and 55 years working in London in 1985–88. In 2002–04 (Phase 7), the genetics study was established and DNA was extracted from 6156 participants. Details of the study design and data collected have been described elsewhere [38].

The English Longitudinal Study of Ageing (ELSA) comprises men and women aged 50 years and over who originally participated in the Health Survey for England in 1998, 1999 or 2001. Fieldwork began in 2002–03 (Phase I) with two-yearly follow-ups and in 2004–05 (Phase II) blood samples were provided by 6231 participants. Details of the cohort have been published elsewhere [39].

The Lothian Birth Cohort 1921 (LBC1921) participants were all born in 1921 and most completed a cognitive ability assessment at age 11 years. In 1999–2001 (Wave I), at approximately 79 years old, 550 participants living in and around Edinburgh, underwent a series of cognitive and physical tests. Details of the recruitment into the study are available on its website (www.lothianbirthcohort.ed.ac.uk) and have been published previously [40], [41].

### Genotyping and Quality Control

Genotype information for *GCKR* rs1260326 (P446L) came from various sources. In NCDS, information came from both the Illumina HumanHap550K v3 and Illumina 1.2 M chips (www.illumina.com) [42]. Data in NSHD and Whitehall II came from the Illumina Metabochip. In ELSA, the closest available proxy was used; SNP rs780094, in very strong linkage disequilibrium (r^2^ = 0.93; 1000 Genomes Pilot 1, CEU population), was obtained via the Applied Biosystems SNPlex 48-plex SNP genotyping system. Information for LBC1921 came from the Illumina Human 610-Quadv1 Chip [43]. Departure from the Hardy-Weinberg equilibrium was assessed in all studies using the chi-square test.

### Phenotypes

#### Anthropometry and biological function

Several measures of anthropometry and biological function were used, where available in the cohorts. Body mass index (BMI kg/m^2^) was calculated as weight divided by height squared derived from measurements conducted at clinics, during a clinical interview in the home, or from self-report. Waist-hip ratio (WHR) was defined as waist circumference (cm) divided by hip circumference (cm). Sitting systolic blood pressure (SBP), diastolic blood pressure (DBP) (mmHg) and pulse rate (BPM) were recorded at the clinical interview; where more than one measurement was taken the mean values were used in analysis. Spirometry was used to assess lung function: forced vital capacity (FVC) and forced expiratory volume (FEV) in 1s (L); the highest value was used in the analyses. Blood samples were used to measure fibrinogen (g/L), total, low-density lipoprotein (LDL) and high-density lipoprotein (HDL) cholesterol (mmol/L), triglycerides (mmol/L), fasting glucose (mmol/L) and non-fasting glycosylated hemoglobin (HbA1c, %).

#### Cognitive capability

A number of cognitive performance tests in the different studies were used to assess cognitive capability. Different assessments of verbal memory were conducted: in ELSA and NCDS, a list of 10 common words were used, with participants asked to recall the list immediately and again after a delay, the mean score was used in the analysis; in NSHD, 15 words were used over three trials; in Whitehall II 20 words were used; responses in NSHD and Whitehall II were given in writing. In Whitehall II, participants recalled in writing in 1 min as many words as possible beginning with ‘S’ to assess phonemic fluency, while in LBC1921 three letters ‘C’, ‘F’ and ‘L’ were used with responses given orally. Participants were asked to recall as many animals as possible within 1 min to measure semantic fluency; responses were given orally in ELSA, NCDS and NSHD, and in writing in Whitehall II. To assess search speed [44], 1-min letter searches among grids of letters were used; 600 letters in NSHD and 780 in ELSA and NCDS.

#### Physical capability and activity

Grip strength was measured in NSHD, ELSA and LBC1921 using electronic or hydraulic dynamometers, with the best measure used in the analysis where more than one trial was conducted. Speeds were calculated from timed walks over 2.44 m (8 feet) and 6 m carried out in ELSA and LBC1921 respectively, with the fastest speed used in the analysis where more than one trial was conducted. Timed chair rises [45] involved asking participants to rise from a chair and sit back down 5 times in ELSA and 10 times in NSHD; the reciprocal of time taken in seconds × 100 [46] was used in the analysis. Standing balance tests were conducted with participants’ eyes open using the Flamingo Balanace Test [47] (stopped at 30 s) in NSHD and the side-by-side, semi-tandem and full tandem [48] in ELSA. Poor standing balance was defined for this analysis as the inability to complete 5 s. Further details of these measurements in these cohorts are presented elsewhere [49]. Levels of physical activity were derived from self-reported levels using questionnaires. Individuals were categorised as ‘physically active’ in this analysis if they engaged in at least moderate sport or activities at least monthly in NCDS, NSHD and LBC1921, or vigorous sport or activities at least monthly in ELSA or at least weekly in Whitehall II.

### Statistical Methods

Where information on ethnicity was collected, non-European participants were excluded from analyses in order to avoid confounding from population stratification [50]. Within studies, linear and logistic regression analyses were conducted on the continuous and dichotomous traits within the cohorts respectively, adjusting for age in the age heterogeneous cohorts of ELSA and Whitehall II, as well as sex. Given the strong associations with triglycerides [12], [13], in a second model, measures were additionally adjusted for it, as well as height and weight, except when testing for the associations with height, weight, BMI and triglycerides. Additive models were used with genotypes coded as 0, 1 and 2 for the number of minor alleles. Likelihood ratio tests were used to compare the fit of the additive models compared with the full genotype models. For continuous traits, the normality of the standardised residuals was inspected with distributional diagnostic plots. For the harmonisation of continuous traits that were used to obtain pooled estimates of the genotypic effects, z-score units were calculated in each study by subtracting the study mean and dividing by its standard deviation. The overall mean for z-scores is 0 and standard deviation 1. Two-step [51] meta-analyses using a random-effects model were performed to obtain pooled genotypic effects. The I^2^ measure was used to quantify heterogeneity [52]. Additionally, meta-analyses of the interaction terms in the second model between the SNP and physical activity, as well as between the SNP and sex were conducted for all outcome measures. Reporting of the analyses met the appropriate items of recommended checklists [53], [54]. A two-tailed significance level of p<0.05 was used as evidence of statistical significance. Statistical analysis was performed in Stata 11.2 (StataCorp LP). Quanto [55] was used for power calculations using the overall minor allele frequency (MAF) of 0.39.

## Results

### Cohort Summaries and Genotyping Quality

Of the 17,004 participants with valid genotypic data, 753 (4.4%) were excluded due to missing values of height, weight and triglycerides, leaving 16,251, as presented in Table 1. Similar genotypic frequencies were observed across the studies and the HWE condition was met for both sources of the NCDS data, as well as for all other studies (p-values>0.07), except NSHD (p-value = 0.03).

**Table 1:** Summary of Sex, Age and *GCKR* Genotype Frequencies by Cohort.

Cohort	Male (%)	Age* in years,	C/C	C/T	T/T	Total
		median (range)	n (%)	n (%)	n (%)	
NCDS	50	44	1901 (36.4)	2513 (48.1)	808 (15.5)	5222
NSHD	47	53	819 (35.8)	1141 (49.8)	329 (14.4)	2289
Whitehall II	77	59 (50–73)	1110 (35.3)	1535 (48.8)	502 (16.0)	3147
ELSA¶	46	64 (52–90+)	1922 (37.7)	2438 (47.8)	736 (14.4)	5096
LBC1921	42	79 (77–80)	195 (39.2)	230 (46.3)	72 (14.5)	497
Total	54	53 (44–90+)	5947 (36.6)	7857 (48.3)	2447 (15.1)	16251

* Age at phase from which the majority of variables are taken, i.e. ELSA: II; LBC1921: I; NCDS: Biomedical Survey (2002); NSHD: 1999 Collection; Whitehall II: VII. ELSA: English Longitudinal Study of Ageing; NCDS: National Child Development Study; NSHD: National Survey of Health and Development.

¶ rs780094 in ELSA, r^2^ = 0.93 with rs1260326.

### Associations between Genotype and Phenotypes

Results of the investigations between the SNP and measures of anthropometry and biological function are presented in Table 2 and Table S1. Evidence of associations in the pooled analyses between the SNP and measures of anthropometry were only observed after adjusting for triglycerides, with the T allele being associated with lower weight and BMI (Figure S1). The T allele was also associated with lower WHR after adjusting for age, sex and triglycerides (p-value = 0.002; data not shown), though this association no longer remained after additional adjustment for height and weight (Table 2). There was no evidence for associations between the *GCKR* genotype and measures of blood pressure or pulse rate from the pooled results of either model (p-values>0.2; Table 2 and Table S1). There was some evidence that the T allele was associated with higher FVC, after additional adjustment for height, weight and triglycerides (Table 2, Table S1, Figure S2), though there was heterogeneity in the associations with FEV. Evidence for an association between the T allele and increased HDL cholesterol was apparent in the second model (Figure S3), with evidence for heterogeneity among the studies. There was very strong evidence for an association between the T allele and higher levels of triglycerides, which strengthened after adjustment for body size (p-value = 3×10^−11^; Figure S4). Associations between the T allele and lower glucose were also stronger in the second model (Figure S5). There was no evidence for associations between the SNP and fibrinogen or LDL cholesterol (p-values>0.1).

**Table 2:** Anthropometry and Biological Function by *GCKR* Genotype (Pooled Results).

		Model 1*			Model 2**		
Variable	Total	Beta (95% CI)	p	I^2%^; Het p	Beta (95% CI)	p	I^2%^; Het p
Height, cm	16251	−0.011 (−0.030–0.007)	0.23	24.6; 0.26	0.006 (−0.011–0.023)	0.48	23.7; 0.26
Weight, kg	16251	−0.018 (−0.047–0.011)	0.23	45.1; 0.12	−0.040 (−0.061–−0.020)	0.0001	15.9; 0.31
BMI, kg/m^2^	16251	−0.011 (−0.041–0.019)	0.48	37.1; 0.17	−0.045 (−0.067–−0.022)	0.0001	7.4; 0.36
Waist-hip ratio	15680	0.006 (−0.011–0.022)	0.51	0.0; 0.51	−0.010 (−0.024–0.004)	0.15	0.0; 0.77
Systolic blood pressure, mmHg	15630	0.007 (−0.027–0.041)	0.67	51.9; 0.08	−0.002 (−0.035–0.032)	0.92	52.8; 0.08
Diastolic blood pressure, mmHg	15630	0.003 (−0.035–0.042)	0.87	61.3; 0.035	−0.003 (−0.041–0.036)	0.90	63.6; 0.027
Pulse rate, BPM	11990	−0.003 (−0.028–0.022)	0.80	0.0; 0.55	−0.015 (−0.039–0.010)	0.24	0.0; 0.42
Forced vital capacity, L	12587	0.013 (−0.006–0.032)	0.19	0.0; 0.64	0.022 (0.005–0.039)	0.013	0.0; 0.49
Forced expiratory volume, L	12588	0.019 (−0.012–0.051)	0.23	51.5; 0.10	0.030 (−0.005–0.065)	0.09	65.0; 0.036
Fibrinogen, g/L	13530	0.020 (−0.011–0.050)	0.21	30.9; 0.23	0.019 (−0.008–0.046)	0.16	15.7; 0.31
Total cholesterol, mmol/L	16251	0.052 (0.019–0.084)	0.0018	47.6; 0.11	0.013 (−0.018–0.045)	0.41	51.2; 0.08
HDL cholesterol, mmol/L	15575	0.000 (−0.037–0.037)	0.98	64.3; 0.038	0.042 (0.003–0.081)	0.033	75.0; 0.007
Log triglycerides, mmol/L	16251	0.105 (0.064–0.147)	6.2×10^−7^	67.4; 0.015	0.112 (0.079–0.145)	2.8×10^−11^	54.9; 0.064
LDL cholesterol, mmol/L	15128	−0.004 (−0.027–0.019)	0.75	0.0; 0.73	−0.018 (−0.041–0.005)	0.12	0.0; 0.90
Glucose¶	14106	−0.042 (−0.066–−0.018)	0.0007	0.0; 0.60	−0.056 (−0.080–−0.033)	2.6×10^−6^	0.0; 0.74

Het- heterogeneity. Beta coefficients per T allele based on z-scores.

* Adjusted for age and sex.

** Additional adjustments: i) height model: weight and triglycerides; ii) weight model: height and triglycerides; iii) triglycerides model: height and weight; iv) BMI model: triglycerides; v) all other models: height, weight and triglycerides.

¶ Glucose in mmol/L or HbA1c in %.

No genotypic associations were observed for any of the cognitive capability traits in the pooled analyses (p-values>0.1; Table 3), or for any of the measures of physical capability (p-values>0.1; Table 4). Additional adjustment for glucose did not materially change the null associations observed for the cognitive and physical capability measures (data not shown).

**Table 3:** Cognitive Capability by Cohort and *GCKR* Genotype.

			Model 1*			Model 2**		
Variable	Cohort	Mean (SD) [n]	Beta (95% CI)	p	I^2^%; Het p	Beta (95% CI)	p	I^2^%; Het p
Word recall- 10 words	NCDS	6.0 (1.5) [5159]	0.00 (−0.04–0.04)	0.85		0.01 (−0.03–0.04)	0.79	
Word recall- 45 words	NSHD	23.9 (6.3) [2235]	0.05 (−0.01–0.12)	0.08		0.07 (0.01–0.13)	0.0324	
Word recall- 20 words	Whitehall II	7.0 (2.4) [3103]	−0.03 (−0.08–0.02)	0.25		−0.02 (−0.07–0.02)	0.33	
Word recall- 10 words	ELSA	5.0 (1.7) [5044]	0.02 (−0.02–0.06)	0.31		0.03 (−0.01–0.06)	0.16	
	**Pooled**	**[15541]**	**0.010 (−0.019–0.038)**	**0.50**	**37.6; 0.19**	**0.016 (−0.016–0.047)**	**0.33**	**48.5; 0.12**
Phonemic fluency- 1 letter	Whitehall II	16.0 (4.1) [3093]	0.03 (−0.02–0.08)	0.20		0.04 (−0.01–0.09)	0.11	
Phonemic fluency- 3 letters	LBC1921	40.5 (12.1) [494]	0.01 (−0.12–0.14)	0.84		−0.00 (−0.13–0.13)	0.99	
	**Pooled**	**[3587]**	**0.030 (−0.016–0.077)**	**0.20**	**0.0; 0.78**	**0.035 (−0.012–0.082)**	**0.14**	**0.0; 0.56**
Semantic fluency	NCDS	22.5 (6.3) [5189]	−0.01 (−0.05–0.03)	0.67		−0.01 (−0.05–0.03)	0.73	
	NSHD	23.7 (6.8) [2275]	0.00 (−0.06–0.06)	0.96		0.01 (−0.05–0.07)	0.80	
	Whitehall II	16.0 (3.7) [3103]	0.00 (−0.05–0.05)	0.90		0.01 (−0.04–0.06)	0.63	
	ELSA	20.3 (6.1) [5047]	0.03 (−0.01–0.07)	0.14		0.03 (−0.00–0.07)	0.08	
	**Pooled**	**[15614]**	**0.008 (−0.014–0.030)**	**0.49**	**0.0; 0.59**	**0.013 (−0.009–0.035)**	**0.26**	**0.0; 0.53**
Search speed- 780 letters	NCDS	333 (86) [5089]	0.01 (−0.03–0.05)	0.60		0.01 (−0.03–0.05)	0.59	
Search speed- 600 letters	NSHD	282 (76) [2264]	0.00 (−0.06–0.06)†	0.95		0.01 (−0.05–0.07)	0.73	
Search speed- 780 letters	ELSA	300 (90) [4990]	0.01 (−0.03–0.05)	0.74		0.01 (−0.03–0.05)	0.58	
	**Pooled**	**[12343]**	**0.007 (−0.018–0.033)**	**0.57**	**0.0; 0.97**	**0.011 (−0.014–0.036)**	**0.39**	**0.0; >0.99**

Het- heterogeneity. Beta coefficients per T allele based on z-scores. NCDS: Sweep 8 (2008).

* Adjusted for age and sex.

** Additionally adjusted for height, weight and triglycerides in all models.

† Full genotype model representing a significantly better fit than the given per allele model.

**Table 4:** Physical Capability by Cohort and *GCKR* Genotype.

			Model 1*			Model 2**		
Variable	Cohort	Mean (SD) [n]	Beta (95% CI)	p	I^2^%; Het p	Beta (95% CI)	p	I^2^%; Het p
Grip strength, kg	NSHD	37.8 (14.4) [2206]	−0.01 (−0.05–0.04)	0.77		0.00 (−0.04–0.05)	0.84	
	ELSA	32.2 (11.5) [5053]	−0.00 (−0.03–0.02)	0.86		0.00 (−0.02–0.03)	0.82	
	LBC1921	26.7 (9.1) [497]	−0.06 (−0.14–0.02)	0.16		−0.06 (−0.14–0.02)	0.14	
	**Pooled**	**[7756]**	−**0.007 (−0.027–0.014)**	**0.53**	**0.0; 0.44**	−**0.002 (−0.025–0.021)**	**0.87**	**11.8; 0.32**
Timed 2.44 m walk, m/s	ELSA	0.94 (0.29) [3222]	0.03 (−0.01–0.08)	0.17		0.05 (−0.00–0.09)	0.05	
Timed 6 m walk, m/s	LBC1921	1.41 (0.37) [494]	0.01 (−0.12–0.13)	0.92		−0.02 (−0.15–0.10)	0.69	
	**Pooled**	**[3716]**	**0.029 (−0.014–0.073)**	**0.19**	**0.0; 0.69**	**0.034 (−0.015–0.084)**	**0.17**	**8.4; 0.30**
Timed chair rises¶-10 rises	NSHD	5.2 (1.7) [2148]	0.01 (−0.05–0.08)	0.69		0.01 (−0.05–0.07)	0.76	
Timed chair rises¶-5 rises	ELSA	9.7 (3.1) [4472]	0.02 (−0.02–0.06)	0.24		0.03 (−0.01–0.07)	0.17	
	**Pooled**	**[6620]**	**0.021 (−0.013–0.054)**	**0.23**	**0.0; 0.77**	**0.022 (−0.011–0.056)**	**0.19**	**0.0; 0.64**
**Variable**	**Cohort**	**Total n (%)** †	**OR (95% CI)**	**p**	**I^2^%; Het p**	**OR (95% CI)**	**p**	**I^2^%; Het p**
Balance <5s-One legged	NSHD	87 (3.9)	1.15 (0.84–1.58)	0.37		1.18 (0.86–1.62)	0.32	
Balance <5s-Tandem	ELSA	573 (11.2)	0.96 (0.84–1.10)	0.58		0.94 (0.81–1.08)	0.35	
	**Pooled**	**[660/7326]**	**0.995 (0.865–1.144)**	**0.94**	**7.9; 0.30**	**1.001 (0.816–1.229)**	**0.99**	**39.7; 0.20**

Het- heterogeneity. Beta coefficients per T allele based on z-scores.

* Adjusted for age and sex.

** Additionally adjusted for height, weight and triglycerides in all models.

¶ Reciprocal of time taken in sec × 100.

† Data within cohorts represent the number (%) of participants unable to balance for at least 5s. Data for pooled row represent the number of participants unable to balance for at least 5s/number of participants who performed the test.

In only a relatively small number of tests did the per allele model represent a significantly poorer fit than the full genotype model (indicated in Table 3 and Table S1).

### Interactions between Genotype and Physical Activity

There was suggestive evidence that physical activity attenuated the negative association between the minor allele and weight towards the null (pooled p-value for interaction terms for weight = 0.07, Figure S6; BMI p-value = 0.1, data not shown) with effects only observed in the physically inactive individuals when analysed separately (pooled beta for weight z-score = −0.06, p-value = 0.0001, n = 8289 in inactive; beta = −0.02, p-value = 0.1, n = 7854 in active; data not shown). There was also suggestive evidence that the raising effects of the minor allele on log-transformed triglycerides was larger among the physically inactive individuals by around 0.04 z-score units (pooled p-value for interaction terms = 0.06; Figure S7). Figure S8 shows evidence for different genotypic effects in the two groups on LDL cholesterol (pooled p-value for interaction terms = 0.04), with a negative effect of the minor allele observed in the physically active group when analysed separately (pooled beta for LDL cholesterol z-score = 0.01, p-value = 0.7, n = 7725 in inactive; beta = −0.04, p-value = 0.008, n = 7364 in active; data not shown). There was no evidence that the genotypic effects on the other measures differed by physical activity status (p-values>0.09, data not shown).

### Interactions between Genotype and Sex

Figures S9 and S10 show that there is evidence that the effects of the genotype on SBP and DBP differed by sex (pooled p-value for interaction terms = 0.002 and 0.005, respectively), with a lowering effect of the minor allele observed in males but not females when analysed separately (e.g. pooled beta for SBP z-score = −0.04, p-value = 0.01, n = 8431 in males; beta = 0.04, p-value = 0.1, n = 7199 in females; data not shown). There was also evidence for an interaction between the genotype and sex on FEV, fibrinogen and phonemic fluency (pooled p-values for interaction terms = 0.04, 0.01 and 0.01, respectively; Figures S11 to S13), though in neither sex did the effects reach statistical significance.

## Discussion

We examined associations between the functional [11] *GCKR* SNP rs1260326 (P446L) and several age-related phenotypes, including previously investigated cardiometabolic biomarkers as well as novel investigations into other phenotypes including measures of cognitive and physical capability, in 16,251 men and women aged between 44 and 90+ years from five UK cohorts. We confirmed the well-reported associations between the minor (T) allele of the SNP and higher levels of triglycerides and lower levels of glucose. In addition, we observed an association between the allele and lower weight and BMI, as well as higher FVC and HDL, which was only apparent after adjusting for triglycerides. A stronger association with glucose was observed after triglyceride adjustment. Despite the reported associations with a range of biomarkers [12], [13], [15]–[20], we found no strong evidence for associations between the SNP and our other age-related phenotypes, including measures of grip strength and verbal memory. Our investigation into interactions between the genotype and self-reported physical activity on our phenotypes provide further evidence for a possible reduction in the genotypic effect on triglycerides in physically active individuals [25], a reduction in triglyceride-adjusted LDL cholesterol in active individuals, as well as evidence for the triglyceride-adjusted effect on weight occurring in physically inactive individuals only. We also found evidence for sex differences in the genotypic effects on SBP and DBP with reduced effects of the T allele only observed in males. These findings may provide insight into the mechanisms of *GCKR* on various phenotypes and should encourage the continued exploration into the modifiers of the genotypic effects of this and other genes.

GWAS and candidate gene studies have identified SNPs in *GCKR* that are strongly associated with a range of traits. In particular, there is very strong evidence that the T allele of rs1260326 is associated with increased levels of triglycerides [12], [13], CRP [15] and lower glucose [10], however there is disagreement among previous reports regarding the associations for some other phenotypes. For instance, many studies including one conducted in 4363 Europeans [21] found no association between the SNP and BMI and WHR, whereas an investigation into 2900 Han Chinese older adults found an association between the T allele and lower BMI and waist circumference [56] which, unlike in our study, was apparent before adjustment for triglycerides. Furthermore, differences have been reported for SNPs in the gene regarding associations with apolipoprotein B [12], [13], LDL [14], [57] and HDL cholesterol [14], 58. With respect to the latter, similar to our findings, a positive association with the minor allele of rs780094 (r^2^ with rs1260326 is 0.93) and HDL cholesterol was observed only after adjusting for triglycerides in a study of around 10,000 middle-aged adults [59]; suggesting the very strong association with triglycerides may be confounding associations between SNPs in the gene and other traits. Our findings for differential genotypic effects on triglycerides, LDL cholesterol and weight by physical activity status are in line with other reports that suggest that physical activity can modify genetic effects [60], [61] and, along with confounding by triglycerides, may provide insight into the contributing factors for the mixed reports for associations of SNPs in *GCKR* on phenotypes. We also found evidence for an interaction between the *GCKR* genotype and sex on SBP and DBP, though, a large multi-study investigation comprising over 34,000 individuals of European ancestry found no association between SNP rs780094 and SBP and DBP [58], however, further investigations into these associations by sex are required to determine whether our findings in males are true associations.

To the best of our knowledge, this is the first report of an investigation between SNPs in *GCKR* and measures of lung function, physical and cognitive capability. The null associations observed for physical and cognitive capability, both before and after adjustment for triglycerides, glucose, and body size, in either sex or physical activity group, from our large multi-cohort study provides evidence against the importance of the gene to ageing beyond cardiometabolic biomarkers and weight. Sample size calculations determined that we were well-powered to detect reasonably small effects on quantitative traits. For example, around 5000 individuals would be required to detect a beta coefficient of 0.06 z-score units with 80% power at the 5% significance level, corresponding to a difference in semantic fluency of around 0.7 points between the two homozygote groups, assuming a standard deviation of 6, or around 1.3 kg in grip strength, assuming a standard deviation of 11. Whilst further larger investigations into the measures of cognitive and physical capability could reveal some associations, our study did not provide evidence for an effect of *GCKR* rs1260326 on these traits. Therefore, *GCKR* appears not to be a common cause of ageing from the phenotypes we investigated and, as yet, there appear to be very few examples of genes that independently influence a range of diverse ageing domains.

As adjusting for triglycerides revealed further or stronger associations for the SNP with some traits, the lack of availability of other biomarkers in our study may mean that we have missed the identification of further associations. For example, a triglyceride-independent effect on CRP has been observed [25] and relationships between inflammatory biomarkers of ageing and cognitive capability have been hypothesised [26]. Therefore, further investigations into cognitive and physical capability in studies that can adjust for other biomarkers may be useful.

## Conclusion

The results of this large multi-cohort study of middle-aged to older adults confirm associations between the functional *GCKR* SNP rs1260326 (P446L) and triglycerides and glucose, suggest an environmentally modifiable effect on triglycerides, LDL and weight, but do not provide evidence for its relevance to other ageing phenotypes, such as grip strength and verbal memory.

## Supporting Information

Figure S1 **Meta-analysis for the Associations between GCKR Genotype and BMI.** Adjusted for age, sex and triglycerides. Coefficients based on z-scores.
(TIF)

Figure S2 **Meta-analysis for the Associations between GCKR Genotype and FVC.** Adjusted for age, sex, height, weight and triglycerides. Coefficients based on z-scores.
(TIF)

Figure S3 **Meta-analysis for the Associations between GCKR Genotype and HDL Cholesterol.** Adjusted for age, sex, height, weight and triglycerides. Coefficients based on z-scores.
(TIF)

Figure S4 **Meta-analysis for the Associations between GCKR Genotype and Log Triglycerides.** Adjusted for age, sex, height and weight. Coefficients based on z-scores.
(TIF)

Figure S5 **Meta-analysis for the Associations between GCKR Genotype and Glucose.** Adjusted for age, sex, height, weight and triglycerides. Coefficients based on z-scores. HbA1c(%) in NCDS, NSHD and LBC1921; glucose (mmol/L) in Whitehall II and ELSA.
(TIF)

Figure S6 **Meta-analysis for the Interaction between GCKR Genotype and Physical Activity on Weight.** Adjusted for age, sex, height and triglycerides. Coefficients based on z-scores. Comparing participants defined as physically active to those physical inactive.
(TIF)

Figure S7 **Meta-analysis for the Interaction between GCKR Genotype and Physical Activity on Log Triglycerides.** Adjusted for age, sex, height and weight. Coefficients based on z-scores. Comparing participants defined as physically active to those physical inactive.
(TIF)

Figure S8 **Meta-analysis for the Interaction between GCKR Genotype and Physical Activity on LDL Cholesterol.** Adjusted for age, sex, height, weight and triglycerides. Coefficients based on z-scores. Comparing participants defined as physically active to those physical inactive.
(TIF)

Figure S9 **Meta-analysis for the Interaction between GCKR Genotype and Sex on Systolic Blood Pressure.** Adjusted for age, height, weight and triglycerides. Coefficients based on z-scores. Comparing females to males.
(TIF)

Figure S10 **Meta-analysis for the Interaction between GCKR Genotype and Sex on Diastolic Blood Pressure.** Adjusted for age, height, weight and triglycerides. Coefficients based on z-scores. Comparing females to males.
(TIF)

Figure S11 **Meta-analysis for the Interaction between GCKR Genotype and Sex on FEV.** Adjusted for age, height, weight and triglycerides. Coefficients based on z-scores. Comparing females to males.
(TIF)

Figure S12 **Meta-analysis for the Interaction between GCKR Genotype and Sex on Fibrinogen.** Adjusted for age, height, weight and triglycerides. Coefficients based on z-scores. Comparing females to males.
(TIF)

Figure S13 **Meta-analysis for the Interaction between GCKR Genotype and Sex on Phonemic Fluency.** Adjusted for age, height, weight and triglycerides. Coefficients based on z-scores. Comparing females to males.
(TIF)

Table S1 **Anthropometry and Biological Function by GCKR Genotype (Full Results).** Het- heterogeneity. Beta coefficients per T allele based on z-scores. *Adjusted for age and sex. **Additionally adjusted for height, weight and triglycerides in all models, except: i) height- weight and triglycerides, ii) weight- height and triglycerides, iii) triglycerides-height and weight. †Full genotype model representing a significantly better fit than the given per allele model. Whitehall II: fibrinogen from Phase V.
(DOC)
