# Supplementary figures and images for: Associations between a Polymorphism in the Pleiotropic GCKR and Age-Related Phenotypes: The HALCyon Programme
Source: PLoS One. 2013 Jul 23;8(7):e70045. doi: 10.1371/journal.pone.0070045 (PMC3720952; doi:10.1371/journal.pone.0070045)

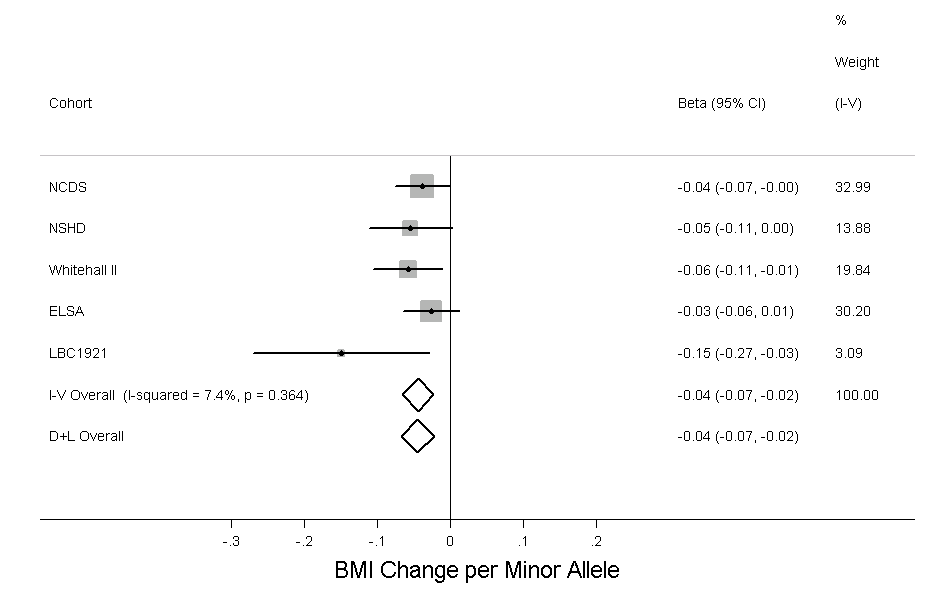

Supplement: Figure S1 — Meta-analysis for the Associations between GCKR Genotype and BMI. Adjusted for age, sex and triglycerides. Coefficients based on z-scores. (TIF) [file pone.0070045.s001.tif]

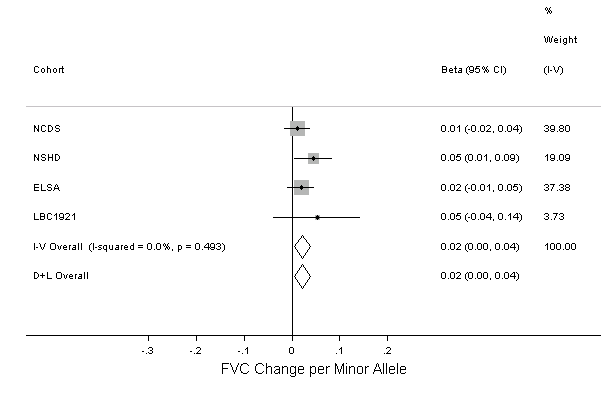

Supplement: Figure S2 — Meta-analysis for the Associations between GCKR Genotype and FVC. Adjusted for age, sex, height, weight and triglycerides. Coefficients based on z-scores. (TIF) [file pone.0070045.s002.tif]

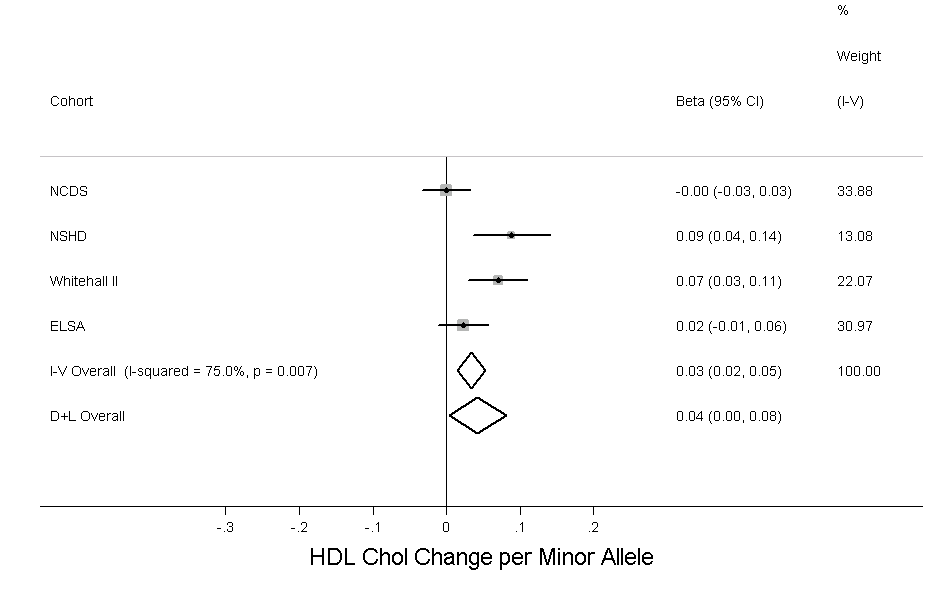

Supplement: Figure S3 — Meta-analysis for the Associations between GCKR Genotype and HDL Cholesterol. Adjusted for age, sex, height, weight and triglycerides. Coefficients based on z-scores. (TIF) [file pone.0070045.s003.tif]

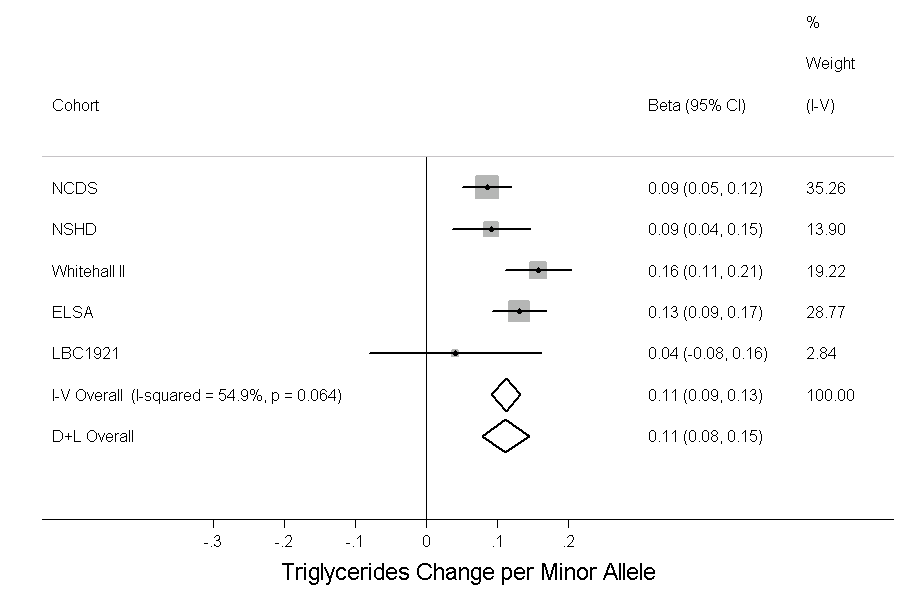

Supplement: Figure S4 — Meta-analysis for the Associations between GCKR Genotype and Log Triglycerides. Adjusted for age, sex, height and weight. Coefficients based on z-scores. (TIF) [file pone.0070045.s004.tif]

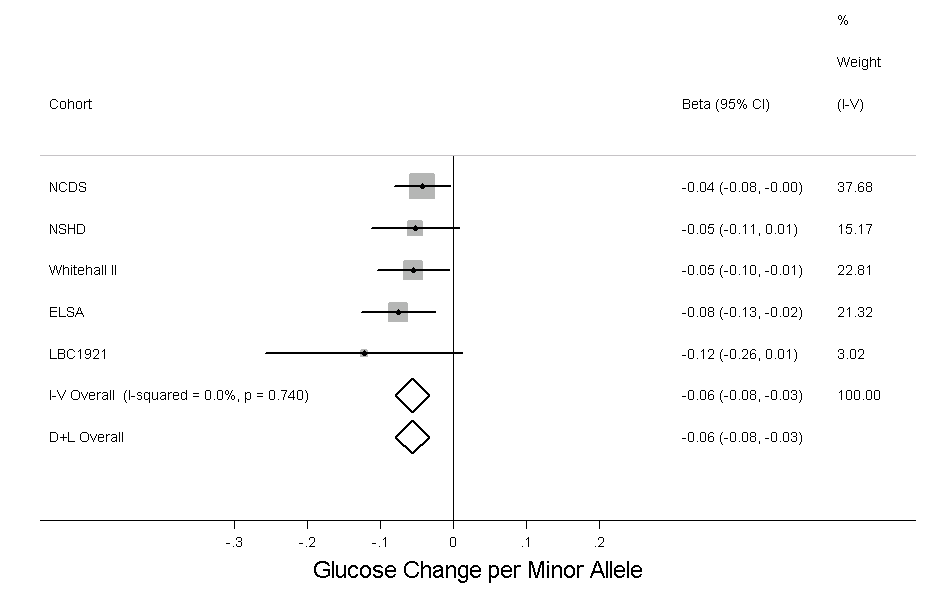

Supplement: Figure S5 — Meta-analysis for the Associations between GCKR Genotype and Glucose. Adjusted for age, sex, height, weight and triglycerides. Coefficients based on z-scores. HbA1c(%) in NCDS, NSHD and LBC1921; glucose (mmol/L) in Whitehall II and ELSA. (TIF) [file pone.0070045.s005.tif]

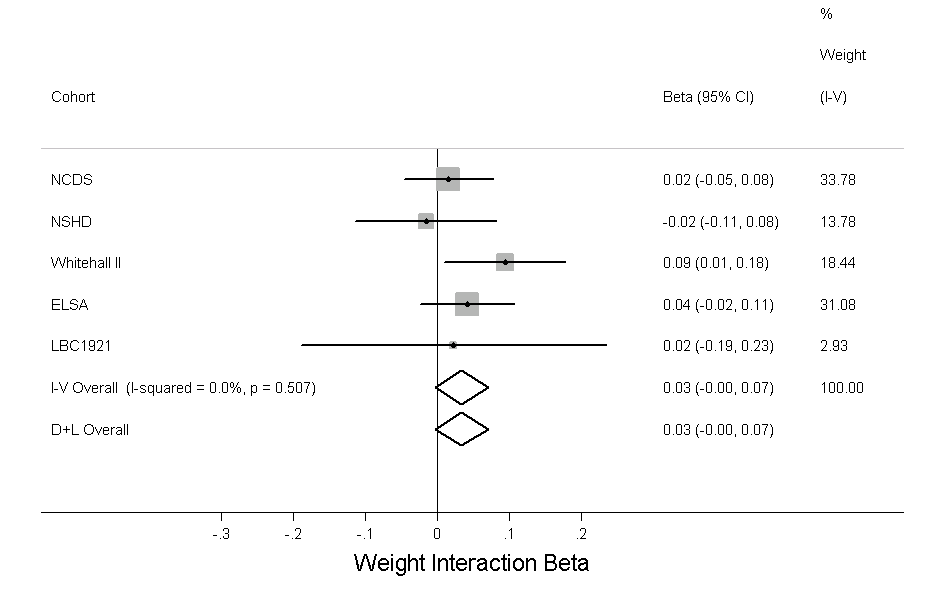

Supplement: Figure S6 — Meta-analysis for the Interaction between GCKR Genotype and Physical Activity on Weight. Adjusted for age, sex, height and triglycerides. Coefficients based on z-scores. Comparing participants defined as physically active to those physical inactive. (TIF) [file pone.0070045.s006.tif]

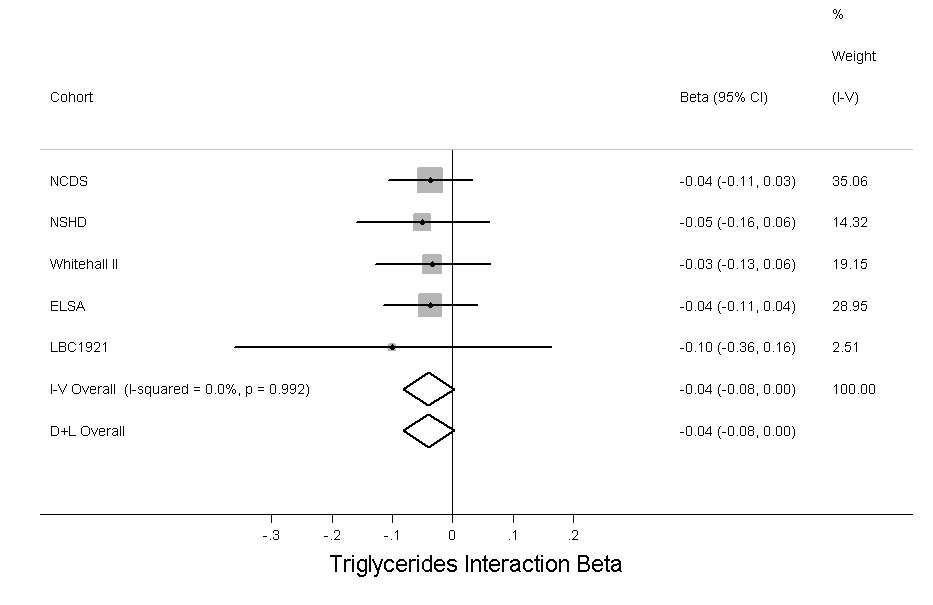

Supplement: Figure S7 — Meta-analysis for the Interaction between GCKR Genotype and Physical Activity on Log Triglycerides. Adjusted for age, sex, height and weight. Coefficients based on z-scores. Comparing participants defined as physically active to those physical inactive. (TIF) [file pone.0070045.s007.tif]

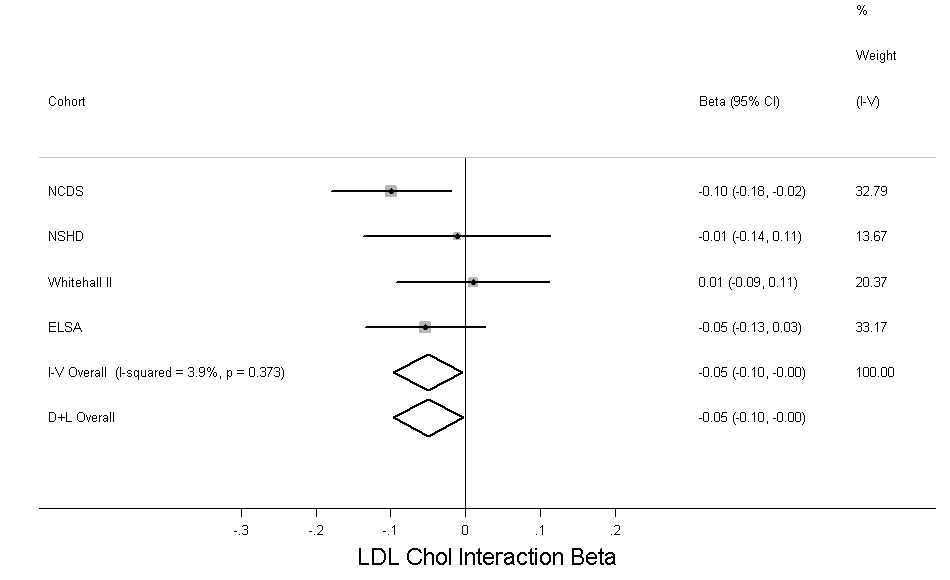

Supplement: Figure S8 — Meta-analysis for the Interaction between GCKR Genotype and Physical Activity on LDL Cholesterol. Adjusted for age, sex, height, weight and triglycerides. Coefficients based on z-scores. Comparing participants defined as physically active to those physical inactive. (TIF) [file pone.0070045.s008.tif]

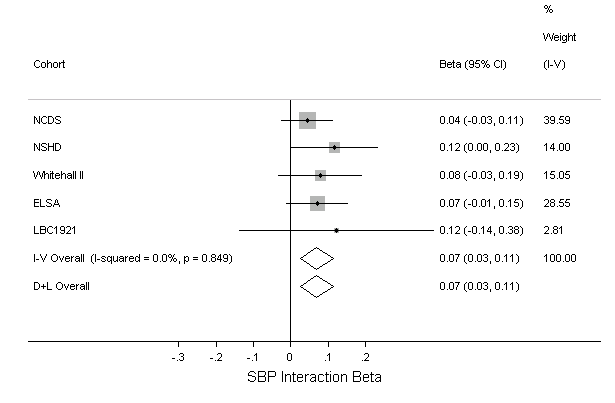

Supplement: Figure S9 — Meta-analysis for the Interaction between GCKR Genotype and Sex on Systolic Blood Pressure. Adjusted for age, height, weight and triglycerides. Coefficients based on z-scores. Comparing females to males. (TIF) [file pone.0070045.s009.tif]

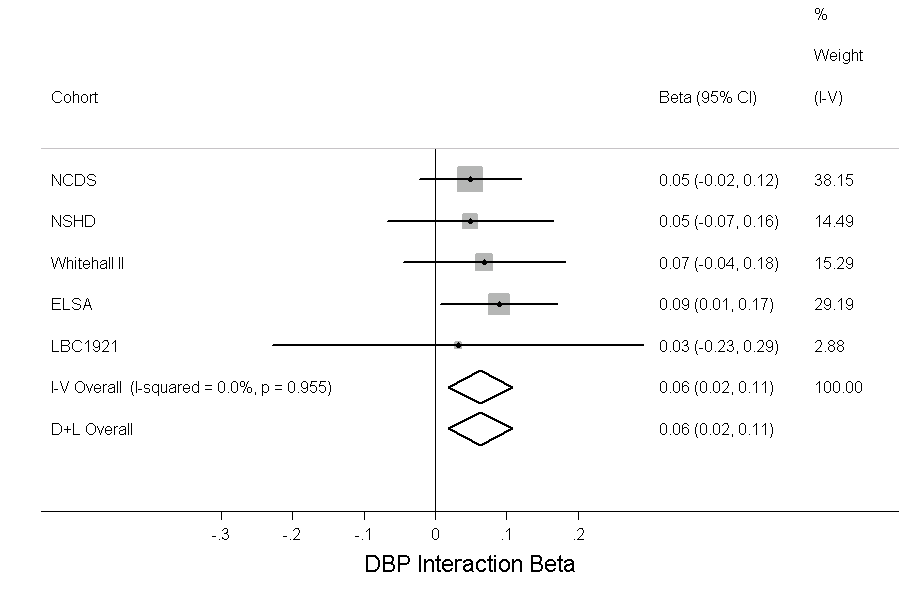

Supplement: Figure S10 — Meta-analysis for the Interaction between GCKR Genotype and Sex on Diastolic Blood Pressure. Adjusted for age, height, weight and triglycerides. Coefficients based on z-scores. Comparing females to males. (TIF) [file pone.0070045.s010.tif]

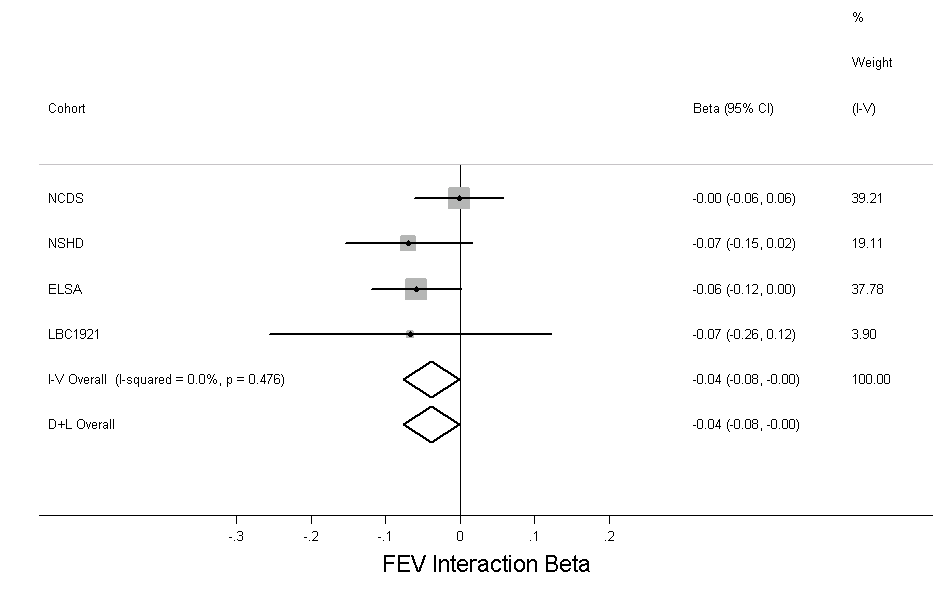

Supplement: Figure S11 — Meta-analysis for the Interaction between GCKR Genotype and Sex on FEV. Adjusted for age, height, weight and triglycerides. Coefficients based on z-scores. Comparing females to males. (TIF) [file pone.0070045.s011.tif]

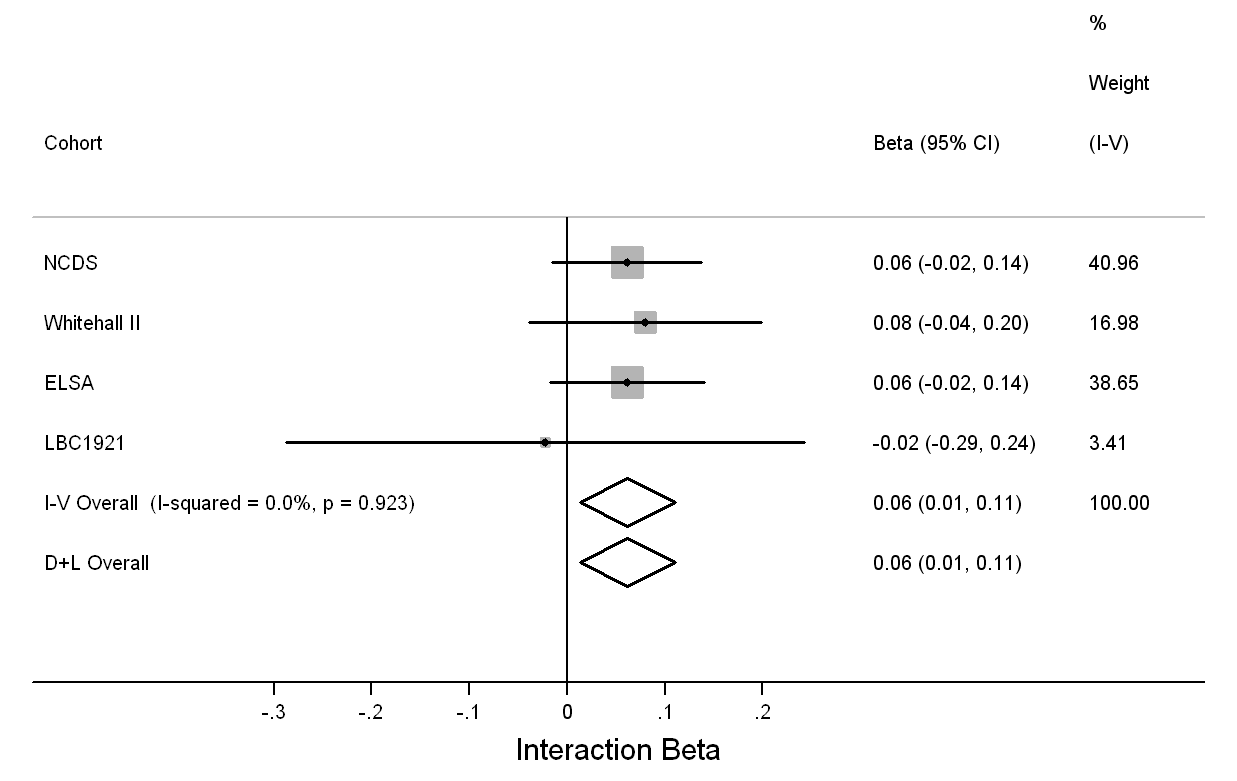

Supplement: Figure S12 — Meta-analysis for the Interaction between GCKR Genotype and Sex on Fibrinogen. Adjusted for age, height, weight and triglycerides. Coefficients based on z-scores. Comparing females to males. (TIF) [file pone.0070045.s012.tif]

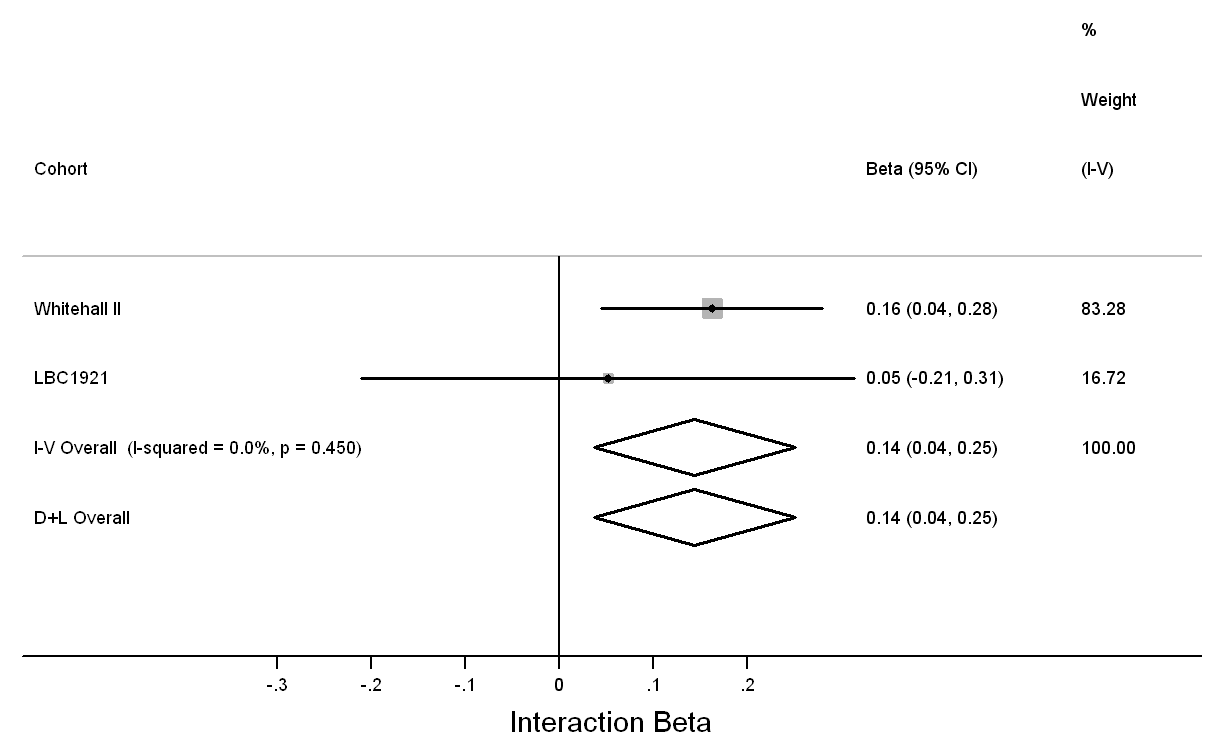

Supplement: Figure S13 — Meta-analysis for the Interaction between GCKR Genotype and Sex on Phonemic Fluency. Adjusted for age, height, weight and triglycerides. Coefficients based on z-scores. Comparing females to males. (TIF) [file pone.0070045.s013.tif]
