# Supplementary material for: Associations between a Polymorphism in the Pleiotropic GCKR and Age-Related Phenotypes: The HALCyon Programme
Source: PLoS One. 2013 Jul 23;8(7):e70045. doi: 10.1371/journal.pone.0070045 (PMC3720952; doi:10.1371/journal.pone.0070045)
Supplement: Table S1 — Anthropometry and Biological Function by GCKR Genotype (Full Results). Het- heterogeneity. Beta coefficients per T allele based on z-scores. *Adjusted for age and sex. **Additionally adjusted for height, weight and triglycerides in all models, except: i) height- weight and triglycerides, ii) weight- height and triglycerides, iii) triglycerides-height and weight. †Full genotype model representing a significantly better fit than the given per allele model. Whitehall II: fibrinogen from Phase V. (DOC) [file pone.0070045.s014.doc]

**Table S1 Anthropometry and Biological Function by *GCKR* Genotype (Full** Results)

|  |  |  | Model 1* |  |  | Model 2** |  |  |
| --- | --- | --- | --- | --- | --- | --- | --- | --- |
| Variable | Cohort | Mean (SD) [n] | Beta (95% CI) | p | I^2^ %; Het p | Beta (95% CI) | p | I^2^ %; Het p |
|  | NCDS | 169.4 (9.3) [5222] | 0.01 (-0.02- 0.03) | 0.69 |  | 0.02 (-0.01- 0.04) | 0.14 |  |
|  | NSHD | 168.2 (8.9) [2289] | -0.05 (-0.09- -0.01) | 0.016 |  | -0.03 (-0.07- 0.01) | 0.17 |  |
| Height, cm | Whitehall II | 172.3 (8.7) [3147] | -0.01 (-0.05- 0.03) | 0.62 |  | 0.01 (-0.02- 0.05) | 0.49 |  |
|  | ELSA | 165.7 (9.4) [5096] | -0.01 (-0.04- 0.02) | 0.39 |  | 0.00 (-0.02- 0.03) | 0.94 |  |
|  | LBC1921 | 163.4 (9.3) [497] | 0.01 (-0.07- 0.10) | 0.77 |  | 0.05 (-0.03- 0.13) | 0.23 |  |
|  | **Pooled** | **[16251]** | **-0.011 (-0.030- 0.007)** | **0.23** | **24.6; 0.26** | **0.006 (-0.011- 0.023)** | **0.48** | **23.7; 0.26** |
|  | NCDS | 78.8 (16.4) [5222] | -0.01 (-0.04- 0.03) | 0.70 |  | -0.03 (-0.06- -0.00) | 0.030 |  |
|  | NSHD | 77.5 (14.8) [2289] | -0.05 (-0.11- 0.00) | 0.07 |  | -0.05 (-0.10- -0.00) | 0.032 |  |
|  | Whitehall II | 79.3 (13.8) [3147] | 0.00 (-0.05- 0.05) | 0.93 |  | -0.04 (-0.09- -0.00) | 0.035 |  |
| Weight, kg | ELSA | 76.4 (15.2) [5096] | 0.00 (-0.03- 0.04) | 0.95 |  | -0.03 (-0.06- 0.01) | 0.13 |  |
|  | LBC1921 | 70.0 (12.6) [497] | -0.13 (-0.24- -0.02) | 0.020 |  | -0.13 (-0.23- -0.03) | 0.008 |  |
|  | **Pooled** | **[16251]** | **-0.018 (-0.047- 0.011)** | **0.2265** | **45.1; 0.12** | **-0.040 (-0.061- -0.020)** | **0.0001** | **15.9; 0.31** |
|  | NCDS | 27.4 (4.9) [5222] | -0.01 (-0.05- 0.03) | 0.67 |  | -0.04 (-0.07- -0.00) | 0.044 |  |
|  | NSHD | 27.4 (4.6) [2289] | -0.02 (-0.08- 0.04) | 0.44 |  | -0.05 (-0.11- 0.00) | 0.060 |  |
|  | Whitehall II | 26.7 (4.2) [3147] | 0.00 (-0.05- 0.05) | 0.97 |  | -0.06 (-0.11- -0.01) | 0.017 |  |
| BMI, kg/m^2^ | ELSA | 27.8 (4.7) [5096] | 0.01 (-0.03- 0.05) | 0.57 |  | -0.03 (-0.06- 0.01) | 0.19 |  |
|  | LBC1921 | 26.2 (4.0) [497] | -0.15 (-0.28- -0.03) | 0.018 |  | -0.15 (-0.27- -0.03) | 0.016 |  |
|  | **Pooled** | **[16251]** | **-0.011 (-0.041- 0.019)** | **0.48** | **37.1; 0.17** | **-0.045 (-0.067- -0.022)** | **0.0001** | **7.4; 0.36** |
|  | NCDS | 0.87 (0.08) [5219] | -0.00 (-0.03- 0.02) | 0.83 |  | -0.01 (-0.04- 0.01) | 0.22 |  |
|  | NSHD | 0.87 (0.09) [2284] | -0.00 (-0.05- 0.04) | 0.84 |  | -0.01 (-0.04- 0.03) | 0.69 |  |
| Waist-hip ratio | Whitehall II | 0.91 (0.09) [3144] | 0.03 (-0.01- 0.07) | 0.11 |  | 0.00 (-0.03- 0.03) | 0.82 |  |
|  | ELSA | 0.89 (0.08) [5033] | 0.01 (-0.02- 0.04) | 0.72 |  | -0.02 (-0.04- 0.01) | 0.23 |  |
|  | **Pooled** | **[15680]** | **0.006 (-0.011- 0.022)** | **0.51** | **0.0; 0.51** | **-0.010 (-0.024- 0.004)** | **0.15** | **0.0; 0.77** |
|  | NCDS | 126.6 (16.6) [5206] | -0.04 (-0.08- -0.00)† | 0.031 |  | -0.05 (-0.08- -0.01)† | 0.009 |  |
|  | NSHD | 136.3 (19.8) [2255] | 0.04 (-0.02- 0.10) | 0.239 |  | 0.03 (-0.03- 0.08) | 0.38 |  |
| Systolic blood pressure, | Whitehall II | 127.7 (16.2) [3145] | 0.01 (-0.04- 0.06) | 0.64 |  | -0.00 (-0.05- 0.04) | 0.85 |  |
| mmHg | ELSA | 134.9 (18.6) [4528] | 0.03 (-0.02- 0.07) | 0.23 |  | 0.02 (-0.03- 0.06) | 0.45 |  |
|  | LBC1921 | 167.8 (26.8) [496] | 0.05 (-0.08- 0.18) | 0.44 |  | 0.06 (-0.07- 0.19) | 0.36 |  |
|  | **Pooled** | **[15630]** | **0.007 (-0.027- 0.041)** | **0.67** | **51.9; 0.08** | **-0.002 (-0.035- 0.032)** | **0.92** | **52.8; 0.076** |
|  | NCDS | 78.8 (10.8) [5206] | -0.05 (-0.09- -0.01)† | 0.006 |  | -0.06 (-0.09- -0.02)† | 0.0026 |  |
|  | NSHD | 84.5 (12.1) [2255] | 0.02 (-0.04- 0.08) | 0.53 |  | 0.02 (-0.04- 0.08) | 0.54 |  |
| Diastolic blood pressure, | Whitehall II | 74.5 (10.4) [3145] | 0.01 (-0.04- 0.06) | 0.67 |  | -0.01 (-0.06- 0.04) | 0.68 |  |
| mmHg | ELSA | 75.3 (11.0) [4528] | 0.03 (-0.02- 0.07) | 0.22 |  | 0.02 (-0.02- 0.06) | 0.32 |  |
|  | LBC1921 | 82.5 (13.0) [496] | 0.06 (-0.07- 0.19) | 0.35 |  | 0.08 (-0.05- 0.21) | 0.23 |  |
|  | **Pooled** | **[15630]** | **0.003 (-0.035- 0.042)** | **0.87** | **61.3; 0.035** | **-0.003 (-0.041- 0.036)** | **0.90** | **63.6; 0.027** |
|  | NCDS | 71.4 (10.6) [5206] | -0.02 (-0.06- 0.02) | 0.41 |  | -0.03 (-0.07- 0.01) | 0.12 |  |
| Pulse rate, BPM | NSHD | 67.9 (10.9) [2256] | -0.01 (-0.07- 0.05) | 0.67 |  | -0.03 (-0.09- 0.03) | 0.39 |  |
|  | ELSA | 59.6 (15.1) [4528] | 0.01 (-0.03- 0.05) | 0.51 |  | 0.00 (-0.03- 0.04) | 0.82 |  |
|  | **Pooled** | **[11990]** | **-0.003 (-0.028- 0.022)** | **0.80** | **0.0; 0.55** | **-0.015 (-0.039- 0.010)** | **0.24** | **0.0; 0.42** |
|  | NCDS | 4.24 (1.05) [5104] | 0.01 (-0.02- 0.04) | 0.54 |  | 0.01 (-0.02- 0.04) | 0.43 |  |
|  | NSHD | 3.50 (0.90) [2206] | 0.02 (-0.02- 0.07) | 0.36 |  | 0.05 (0.01- 0.09) | 0.026 |  |
| Forced vital capacity, L | ELSA | 3.26 (1.08) [4780] | 0.01 (-0.02- 0.04) | 0.68 |  | 0.02 (-0.01- 0.05) | 0.19 |  |
|  | LBC1921 | 2.40 (0.74) [497] | 0.07 (-0.03- 0.17) | 0.16 |  | 0.05 (-0.04- 0.14) | 0.26 |  |
|  | **Pooled** | **[12587]** | **0.013 (-0.006- 0.032)** | **0.19** | **0.0; 0.64** | **0.022 (0.005- 0.039)** | **0.013** | **0.0; 0.49** |
|  | NCDS | 3.28 (0.87) [5104] | -0.00 (-0.04- 0.03) | 0.87 |  | -0.00 (-0.03- 0.03) | 0.95 |  |
|  | NSHD | 2.80 (0.70) [2207] | 0.04 (-0.01- 0.08) | 0.13 |  | 0.06 (0.02- 0.10) | 0.005 |  |
| Forced expiratory volume, L | ELSA | 2.37 (0.85) [4780] | 0.00 (-0.03- 0.04) | 0.78 |  | 0.01 (-0.02- 0.04) | 0.39 |  |
|  | LBC1921 | 1.89 (0.63) [497] | 0.11 (0.02- 0.21) | 0.023 |  | 0.10 (0.01- 0.20) | 0.032 |  |
|  | **Pooled** | **[12588]** | **0.019 (-0.012- 0.051)** | **0.23** | **51.5; 0.10** | **0.030 (-0.005- 0.065)** | **0.09** | **65.0; 0.036** |
|  | NCDS | 2.95 (0.62) [5073] | 0.03 (-0.01- 0.07) | 0.13 |  | 0.03 (-0.00- 0.07) | 0.08 |  |
|  | Whitehall II | 3.00 (0.60) [2948] | 0.04 (-0.01- 0.09) | 0.14 |  | 0.02 (-0.03- 0.07) | 0.36 |  |
| Fibrinogen, g/L | ELSA | 3.22 (0.73) [5037] | 0.01 (-0.03- 0.05) | 0.52 |  | 0.01 (-0.03- 0.05) | 0.52 |  |
|  | LBC1921 | 3.60 (0.90) [472] | -0.10 (-0.23- 0.03) | 0.12 |  | -0.09 (-0.22- 0.04) | 0.16 |  |
|  | **Pooled** | **[13 530]** | **0.020 (-0.011- 0.050)** | **0.21** | **30.9; 0.23** | **0.019 (-0.008- 0.046)** | **0.16** | **15.7; 0.31** |
|  | NCDS | 5.88 (1.09) [5222] | 0.02 (-0.02- 0.06) | 0.37 |  | -0.02 (-0.05- 0.02) | 0.38 |  |
|  | NSHD | 6.09 (1.07) [2289] | 0.10 (0.04- 0.17) | 0.0007 |  | 0.07 (0.01- 0.12) | 0.021 |  |
| Total cholesterol, mmol/L | Whitehall II | 5.70 (1.00) [3147] | 0.07 (0.02- 0.12) | 0.011 |  | 0.01 (-0.03- 0.06) | 0.58 |  |
|  | ELSA | 5.92 (1.20) [5096] | 0.03 (-0.01- 0.07) | 0.13 |  | -0.01 (-0.05- 0.03) | 0.64 |  |
|  | LBC1921 | 5.65 (1.11) [497] | 0.10 (-0.02- 0.22)† | 0.11 |  | 0.08 (-0.03- 0.20)† | 0.17 |  |
|  | **Pooled** | **[16251]** | **0.052 (0.019- 0.084)** | **0.002** | **47.6; 0.11** | **0.013 (-0.018- 0.045)** | **0.41** | **51.2; 0.08** |
|  | NCDS | 1.56 (0.39) [5211] | -0.03 (-0.07- 0.01) | 0.13 |  | -0.00 (-0.03- 0.03) | 0.98 |  |
|  | NSHD | 1.67 (0.48) [2123] | 0.07 (0.01- 0.13) | 0.022 |  | 0.09 (0.04- 0.14) | 0.0009 |  |
| HDL cholesterol, mmol/L | Whitehall II | 1.56 (0.44) [3147] | 0.00 (-0.04- 0.05) | 0.90 |  | 0.07 (0.03- 0.11) | 0.0006 |  |
|  | ELSA | 1.53 (0.39) [5094] | -0.02 (-0.06- 0.02)† | 0.27 |  | 0.02 (-0.01- 0.06) | 0.18 |  |
|  | **Pooled** | **[15575]** | **0.000 (-0.037- 0.037)** | **0.98** | **64.3; 0.038** | **0.042 (0.003- 0.081)** | **0.033** | **75.0; 0.007** |
|  | NCDS | 0.53 (0.60) [5222] | 0.08 (0.05- 0.12) | 1.3x10^-5^ |  | 0.09 (0.05- 0.12) | 1.1x10^-6^ |  |
|  | NSHD | 0.59 (0.57) [2289] | 0.08 (0.02- 0.14) | 0.005 |  | 0.09 (0.04- 0.15) | 0.001 |  |
| Log triglycerides, mmol/L | Whitehall II | 0.19 (0.50) [3147] | 0.16 (0.11- 0.21) | 2.9 x10^-10^ |  | 0.16 (0.11- 0.21)† | 3.5x10^-11^ |  |
|  | ELSA | 0.45 (0.51) [5096] | 0.14 (0.10- 0.18)† | 3.4 x10^-11^ |  | 0.13 (0.09- 0.17) | 1.4x10^-11^ |  |
|  | LBC1921 | 0.52 (0.41) [497] | -0.01 (-0.14- 0.11) | 0.84 |  | 0.04 (-0.08- 0.16) | 0.50 |  |
|  | **Pooled** | **[16251]** | **0.105 (0.064- 0.147)** | **6.2x10^-7^** | **67.4; 0.015** | **0.112 (0.079- 0.145)** | **2.8x10^-11^** | **54.9; 0.064** |
|  | NCDS | 3.41 (0.91) [4938] | -0.01 (-0.05- 0.03) | 0.47 |  | -0.02 (-0.06- 0.02) | 0.25 |  |
|  | NSHD | 3.52 (0.96) [2117] | 0.02 (-0.05- 0.08) | 0.57 |  | 0.00 (-0.06- 0.07) | 0.89 |  |
| LDL cholesterol, mmol/L | Whitehall II | 3.52 (0.92) [3107] | 0.01 (-0.04- 0.06) | 0.64 |  | -0.02 (-0.07- 0.03) | 0.41 |  |
|  | ELSA | 3.59 (1.00) [4966] | -0.01 (-0.05- 0.03) | 0.57 |  | -0.02 (-0.06- 0.02) | 0.33 |  |
|  | **Pooled** | **[15128]** | **-0.004 (-0.027- 0.019)** | **0.75** | **0.0; 0.73** | **-0.018 (-0.041- 0.005)** | **0.12** | **0.0; 0.90** |
| HbA1c, % | NCDS | 5.2 (0.7) [5166] | -0.03 (-0.07- 0.01) | 0.10 |  | -0.04 (-0.08- -0.00) | 0.029 |  |
| HbA1c, % | NSHD | 5.7 (0.7) [2270] | -0.05 (-0.11- 0.02) | 0.15 |  | -0.05 (-0.11- 0.01) | 0.09 |  |
| Glucose, mmol/L | Whitehall II | 5.4 (0.9) [3146] | -0.03 (-0.08- 0.02) | 0.29 |  | -0.05 (-0.10- -0.01) | 0.029 |  |
| Glucose, mmol/L | ELSA | 5.0 (0.9) [3085] | -0.05 (-0.11- -0.00) | 0.036 |  | -0.08 (-0.13- -0.02) | 0.004 |  |
| HbA1c, % | LBC1921 | 5.7 (0.7) [439] | -0.14 (-0.27- -0.00)† | 0.046 |  | -0.12 (-0.26- 0.01)† | 0.08 |  |
|  | **Pooled** | **[14106]** | **-0.042 (-0.066- -0.018)** | **0.0007** | **0.0; 0.60** | **-0.056 (-0.080- -0.033)** | **2.6x10^-6^** | **0.0; 0.74** |

Het- heterogeneity. Beta coefficients per T allele based on z-scores.

*Adjusted for age and sex. **Additionally adjusted for height, weight and triglycerides in all models, except: i) height- weight and triglycerides, ii) weight- height and triglycerides, iii) triglycerides-height and weight.

†Full genotype model representing a significantly better fit than the given per allele model.

Whitehall II: fibrinogen from Phase V.
